# Combined use of gastric pull-up and pectoralis major flaps for massive defects after total laryngopharyngoesophagectomy in patients with advanced hypopharyngeal carcinoma

**DOI:** 10.1007/s00405-014-3358-y

**Published:** 2014-12-09

**Authors:** Caiyun Zhang, Shicai Chen, Minhui Zhu, Donghui Chen, Hezhong Chen, Hongliang Zheng

**Affiliations:** 1 Department of Otorhinolaryngology-Head and Neck Surgery, Changhai Hospital, Second Military Medical University, No.168 Changhai Road, 200433 Shanghai, China; 2 Department of Cardiothoracic Surgery, Changhai Hospital, Second Military Medical University, No.168 Changhai Road, 200433 Shanghai, China

**Keywords:** Hypopharyngeal cancer, Radical surgery, Gastric pull-up, Pectoralis major flap

## Abstract

Reconstruction for patients with advanced squamous cell carcinoma of the hypopharynx (SCCHP) after radical surgery is a challenge for head and neck surgeons, especially when one flap alone cannot entirely cover the defects. In this report, we describe the successful use of gastric pull-up combined with pectoralis major flaps for single-stage reconstruction after total laryngopharyngoesophagectomy in patients with SCCHP. We retrospectively reviewed the records of 23 patients with stage IV SCCHP who underwent this reconstructive procedure. Surgical details and perioperative morbidity were described, and functional and oncologic outcomes were evaluated. We used the gastric pull-up and pectoralis major flap procedure to reconstruct the defects for all 23 patients. In 13 patients the combined flaps were used to restore intestinal continuity, and in 10 patients the defects were repaired using gastric pull-up alone and covered by the pectoralis major flap. All the combined flaps worked well, and patients recovered normal swallowing function a mean 19.6 days after surgery. After an overall mean follow-up time of 25.3 months, six patients were still alive at the time of this analysis with no evidence of disease. Our results indicate that for patients with advanced SCCHP after total laryngopharyngoesophagectomy, using a pectoralis major flap combined with gastric pull-up enables one-stage reconstruction even when gastric pull-up alone cannot restore intestinal continuity. Furthermore, the functional and oncologic outcomes from this study suggest that this reconstructive procedure is safe and reliable, and more patients with advanced disease could be considered.

## Introduction

Squamous cell carcinoma of the hypopharynx (SCCHP), a classification that includes tumors of the piriform sinus, postcricoid area, and posterior pharyngeal wall, is an aggressive cancer in the head and neck region [1]. SCCHP is usually diagnosed at an advanced stage because of its absence of symptoms in the early stages and its early locoregional metastasis due to the area’s rich lymphatic drainage [2]. Therefore, most patients with advanced SCCHP have disease in regional lymph nodes at presentation. Treatment of patients with advanced SCCHP with chemoradiation is effective in preserving laryngeal function without compromising the survival rates obtained previously by primary surgery. In hypopharyngeal cancer, larynx-preserving therapies have been shown to preserve larynx function in about half of the patients at 3–5 years. However, in patients who failed chemoradiation or present with residual disease or develop recurrent disease, palliative treatments will result in moderately short overall survival durations with poor quality of life [3, 4]. Thus, surgical treatment should be considered for patients with those advanced SCCHP to return them to a disease-free state [5–7].

Surgical treatment for patients with advanced hypopharyngeal cancer is a challenge for head and neck surgeons, particularly when the cervical esophagus is involved [8], because of the technical difficulties in restoring digestive continuity after successfully removing the disease. Many reconstructive options have been recommended to repair the massive defects resulting from radical resection [9–13], and the advantages and disadvantages of each method have been comprehensively discussed in the literature [14].

Gastric pull-up, which was firstly described by Shefts and Fischer in 1949, is a standard reconstructive method for patients with advanced hypopharyngeal cancer after total laryngopharyngoesophagectomy [15]. The advantages of gastric pull-up include one-stage closure, single anastomosis, and a reliable blood supply; however, the disadvantages of this technique should also be considered, which include a high morbidity rate, a high perioperative mortality rate, and potential for inadequate tissue for reconstruction because of variations in stomach size and/or extra resection to obtain negative surgical margins [16, 17]. One-stage reconstruction with insufficient stomach tissue creates tension that can lead to anastomotic dehiscence. For this reason, an additional flap is required to ensure a tension-free anastomosis when insufficient tissue is available for gastric pull-up.

In this retrospective analysis of 23 advanced SCCHP patients with cervical esophagus involved or with a synchronous second primary tumor in the thoracic esophagus, we report the reconstruction of the defects using combined gastric pull-up and pectoralis major flaps after total laryngopharyngoesophagectomy and bilateral neck dissection. We also evaluate the perioperative morbidity and functional and oncologic outcomes of these patients.

## Materials and methods

For this analysis, we identified 23 patients with advanced hypopharyngeal cancer who underwent total laryngopharyngoesophagectomy and reconstruction of the defects using gastric pull-up and pectoralis major flaps at our institute from March 2002 to July 2010. All subjects signed an informed consent form that was approved by the Institutional Review Board of the Second Military Medical University. All patients had histopathologically confirmed, previously treated or untreated SCCHP. Clinical data such as the index tumor’s stage at presentation, treatment, main signs or symptoms were obtained from the medical records. Also obtained were postoperative data, including the pathological stage of each tumor, functional outcomes, postoperative outcomes, postoperative therapies, and disease state at last visit.

The clinical stage of each patient’s disease was determined by clinical examination; ultrasonography; magnetic resonance imaging (MRI) or computed tomography scans of the cervical region, thorax, and liver; and laryngoscopy with biopsy of the tumor for histologic confirmation. Gastroscopies and barium meal examinations were also performed to detect second primary cancers in the upper gastrointestinal area and to evaluate the size of the stomach [18]. In addition, ultrasonography was used to locate the pectoral branch of the thoracoacromial artery.

This surgery was performed using a 2-team procedure. Head and neck surgeons performed the primary tumor resection, which consisted of total laryngopharyngoesophagectomy and bilateral neck dissection from level II to level V. Additional resections, including partial thyroidectomy, tonsillectomy, and level VII lymph node dissection, were carried out if positive margins were found by intraoperative frozen-section analysis. A team of thoracic surgeons freed the stomach for reconstruction through an upper midline incision. Care was taken to preserve the right gastric and right gastroepiploic vessels to maintain blood supply of the gastric flap. The stomach was transected at the esophagogastric junction, and the esophagus was moved away from the adjacent thoracic structures. After removal of the malignant tissue, the stomach was then pulled up through the posterior mediastinum into the neck. Due to the massive defect after radical resection, a pectoralis major flap was raised as the last step either to restore the intestinal continuity in combination with the gastric flap or to cover the pharyngogastric anastomosis and the exposed great vessels of the neck.

After wound closure, patients were transferred to an intensive care unit for a short period of time (usually 1–2 days). In the intensive care unit, the patients were monitored by laryngoscopy for early detection of flap failure. Other postoperative care included the administration of antibiotics and analgesics, inspection of the neck at least once a day, and monitoring of vital signs. Before patients began to take food or liquids orally, gastric radiography was performed to detect leakage and to assess swallowing function. If the test showed no evidence of fistula, the nasogastric tube was then removed, and the patients began to receive adjuvant cancer treatment.

Patients were monitored through their treatment and posttreatment course at 3-month intervals during the first 2 years and at 6-month intervals after 2 years. The follow-up examinations included complete clinical examination, endoscopy, chest radiography, abdominal and cervical ultrasonography, and computed tomography scans of the neck. Additional examinations, such as positron emission tomography scans, were conducted when indicated. Patients were considered have no evidence of disease if absence of disease was documented at the date of the last visit with the head and neck surgeon.

## Results

The characteristics of patients before radical surgery are presented in Table 1. All patients were male; their mean age was 56.7 years (range 45–66 years). Histological examination of the biopsy tissues showed that all the cancers were squamous cell carcinoma. Majority of patients had the symptom of dysphagia, as well as other symptoms, such as weight loss, hoarseness, and pain. Nine patients received preoperative treatments, including induction chemotherapy, radiotherapy, laryngectomy, and neck dissection.

**Table 1:** Demographic characteristics of patients with SCCHP (*N* = 23)

Variable	No. patients (%)
Age (year)	
≤55	7 (30.4 %)
>55	16 (69.6 %)
Sex	
Male	21 (91.3 %)
Female	2 (8.7 %)
Smoking	
Never	3 (13.0 %)
Ever	20 (87.0 %)
Alcohol use	
Never	5 (21.7 %)
Ever	18 (78.3 %)

**Fig. 1:**
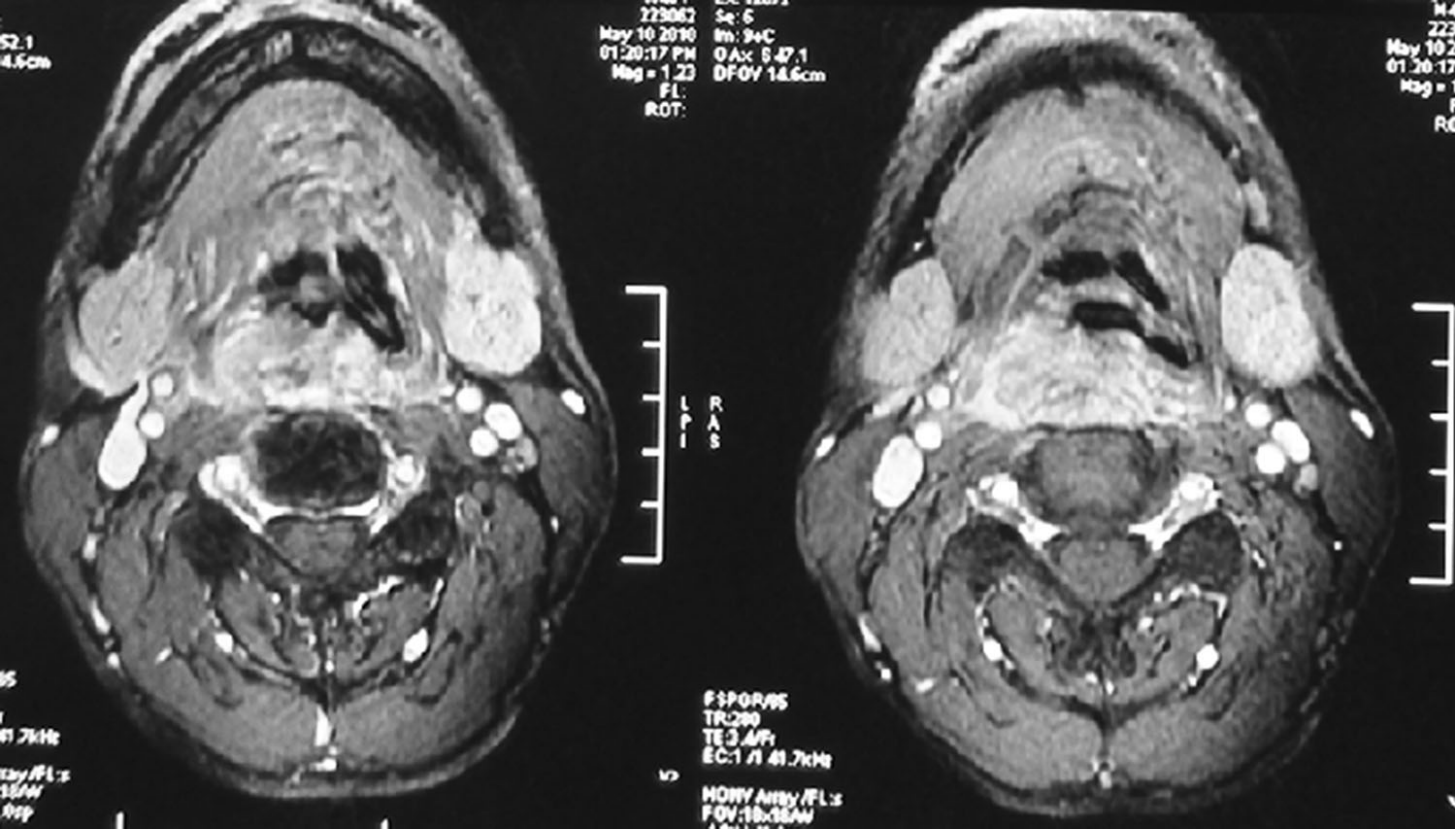
Preoperative MRI examination in a patient with a right piriform sinus lesion involving the cervical esophagus and oropharynx

Table 2 shows each patient’s primary tumor location(s) and the preoperative clinical TNM stage. Preoperative examinations revealed the primary tumor in the postcricoid area in 9 patients and the piriform sinus in 14 patients. All but 3 of these tumors involved the cervical esophagus. In these 23 patients, gastroscopy and barium meal examinations detected a synchronous second primary tumor in the thoracic esophagus in 8 patients. Of the 23 patients, 4 had stage IVB disease, and 19 had stage IVA disease. Figure 1 shows the result of MRI examination in a patient with left piriform sinus lesion involving the cervical esophagus.

**Table 2:** Clinical characteristics of patients with SCCHP (*N* = 23)

Variable	No. patients (%)
Primary tumor location	
PC	9 (39.1 %)
PS	14 (60.9 %)
Cervical esophagus involved	
Yes	19 (82.6 %)
No	3 (17.4 %)
Thoracic esophageal cancer	
Yes	8 (34.8 %)
No	15 (65.2 %)
Stage	
IVA	19 (82.6 %)
IVB	4 (17.4 %)
Treatment	
Surgery only	3 (13.0 %)
Surgery + X/C	20 (87.0 %)

*PC* postcricoid area, *PS* piriform sinus, *X* radiotherapy, *C* chemotherapy

Surgical details are described in Table 3. After total laryngopharyngoesophagectomy and bilateral neck dissection from level II to level V, additional resections were performed, and all patients were reconstructed with gastric pull-up and pectoralis major flaps. As shown in Table 3, pectoralis major flaps were harvested from the right side in 13 patients and from the left side in 10. Of the 23 pectoralis major flaps, 13 were used together with the gastric pull-up flap to restore intestinal continuity, and the rest were used only to cover the neck defects to protect the exposed great vessels and the pharyngogastric anastomoses.

**Table 3:** Surgical details and functional outcomes

Variable	No. patients (%)
Side of PMF raised	
Left	10 (43.5 %)
Right	13 (56.5 %)
PMF use for reconstruction	
Restore intestinal continuity	13 (56.5 %)
Cover the neck defect	10 (43.5 %)
Complications	
None	10 (43.5 %)
Wound infection	8 (34.8 %)
Anastomotic leakage	2 (8.7 %)
Anastomotic stenosis	3 (13.0 %)
Time to swallow, *d*	
≤15	9 (39.1 %)
>15	14 (60.9 %)

*PMF* pectoralis major flap

**Fig. 2:**
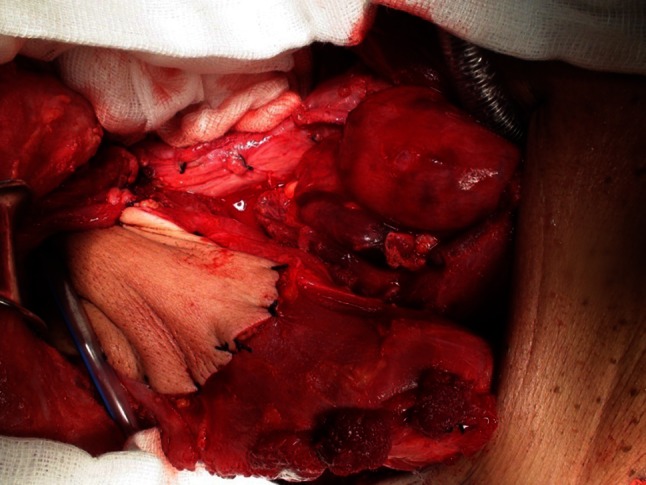
Restoration of intestinal continuity using gastric pull-up and pectoralis major flaps. The stomach has been pulled up into the neck, and a pectoralis major flap has been raised and anastomosed to the posterior pharyngeal wall

**Fig. 3:**
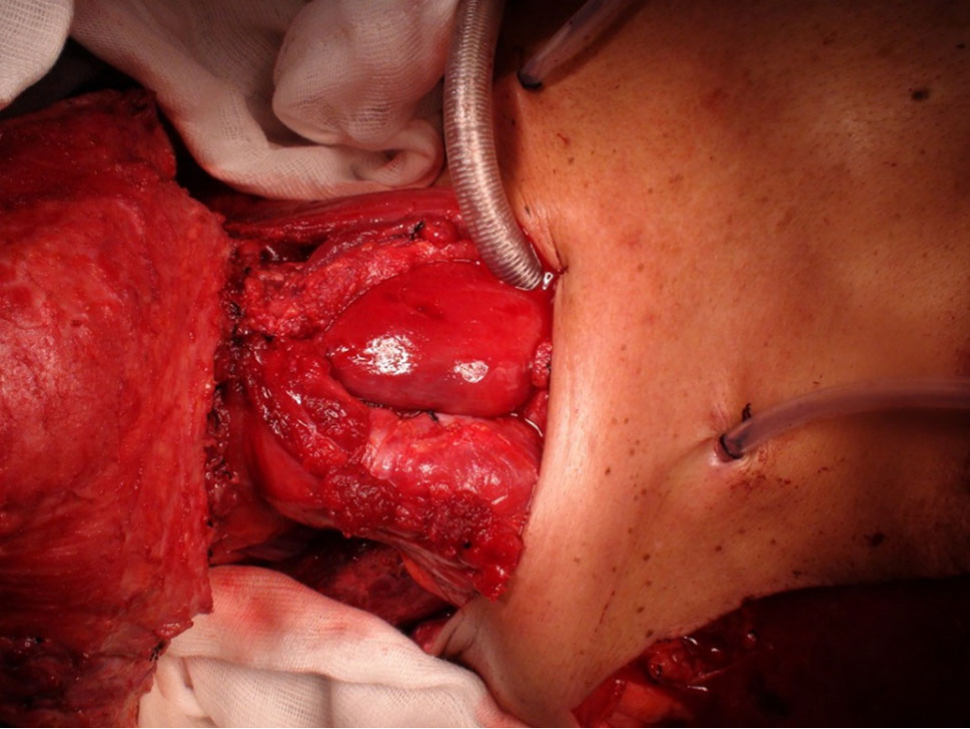
Restoration of intestinal continuity using gastric pull-up and pectoralis major flaps. An inferiorly based flap is created from the surface of the stomach. The distal end of the flap is then sutured to the tongue base and the proximal end to the skin of the pectoralis major flap

As shown in Fig. 2, the pectoralis major flap was anastomosed to the posterior pharyngeal wall, and the gastric flap was pulled up into the neck. Then, an inferiorly based flap was created from the surface of the stomach; the distal end of this flap was sutured to the tongue base and the proximal end to the skin of the pectoralis major flap (Fig. 3).

Thirteen of the 23 patients developed postoperative complications (Table 3). Two patients developed anastomotic leakage, which might have been due to preoperative radiotherapy. The leakage was resolved by surgical intervention using the contralateral pectoralis major flap. Other patients experienced complications that included wound infection and anastomotic stenosis; and none of these complications required surgical intervention. None of the 23 patients experienced flap failure or perioperative death.

**Fig. 4:**
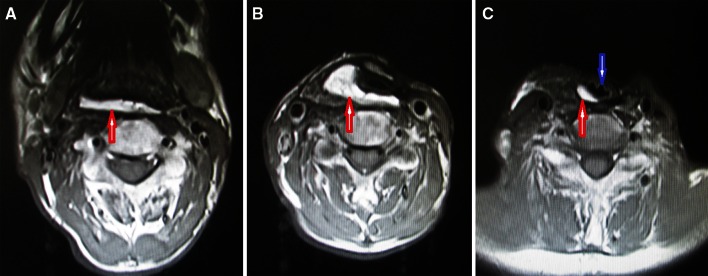
Results of postoperative MRI examination in the same patient following a total laryngopharyngoesophagectomy to remove a left piriform sinus lesion involving the cervical esophagus and reconstruction with gastric pull-up and pectoralis major flaps

**Fig. 5:**
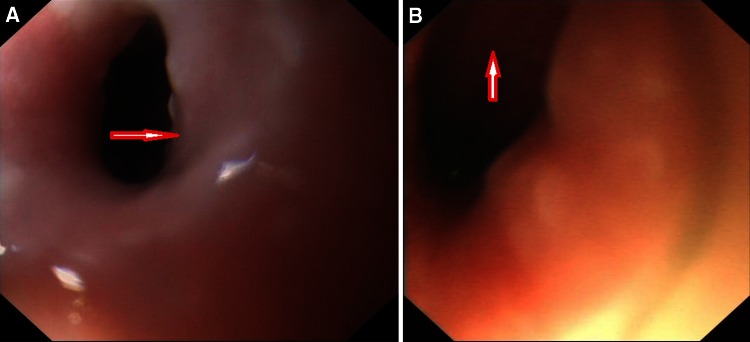
Postoperative endoscopy shows the normal condition of the gastric pull-up and pectoralis major flaps

Swallowing function was evaluated for patients after surgery. Figure 4 shows the results of postoperative MRI examination in the same patient following a total laryngopharyngoesophagectomy to remove a left piriform sinus lesion involving the cervical esophagus and reconstruction with gastric pull-up and pectoralis major flaps, and Fig. 5 shows the normal condition of the combined flaps on endoscopy in the same patient. Of the 23 patients, 9 patients began oral feeding in less than 15 days after surgery, and the rest began oral feeding in more than 15 days after surgery, with a mean swallowing function recovery time of 19.6 days (range 12–28 days) after reconstruction. Furthermore, we found a mean swallowing recovery time of 19.2 days (range 12–25 days) in the patients for whom the pectoralis major flap was used to restore intestinal continuity and a mean swallowing recovery time of 20 days (range 14–28 days) for patients in whom the pectoralis major flap was used to cover the neck. However, because of the small number of patients in each group, we did not conduct a statistical test to determine whether the difference in time to swallowing function recovery was significant.

After radical surgery, all 23 patients received postoperative treatments, which included radiotherapy, chemotherapy and radiotherapy given separately, and concurrent chemoradiation. After an overall mean follow-up time of 25.3 months (range 8–55 months), five patients died of disease less than 12 months after surgery, from primary tumor recurrence or from locoregional metastasis. Three of these patients had pathological grade 2 disease, while the other two patients had grade 3 disease. These five patients had common characteristics of having pT4 tumors and pN2 node disease. Six other patients also had pT4 tumors and pN2 node diseases; however, they were still alive with no evidence of disease at their last follow-up visit. The remaining 12 patients had a similar pathological stage—pT4 tumors and pN1 node disease—however, the oncologic outcomes of these patients varied, with a survival time after surgery from 12 to 36 months (Table 4).

**Table 4:** Pathological stage and oncologic outcomes

Variable	No. patients (%)
Pathological grade	
G1	10 (43.5 %)
G2	11 (47.8 %)
G3	2 (8.7 %)
pT category	
≤T3	0 (0 %)
T4	23 (100 %)
pN category	
N1	12 (52.2 %)
N2	11 (47.8 %)
N3	0 (0 %)
Oncologic outcome (duration, m)	
<12	5 (21.7 %)
12–36	12 (52.2 %)
>36	6 (26.1 %)

*G1* grade 1 (well differentiated), *G2* grade 2 (moderately differentiated), *G3* grade 3 (poorly differentiated)

## Discussion

In this retrospective study of 23 patients with advanced SCCHP, 8 of whom had synchronous second primary tumor in the thoracic esophagus, we reported a successful reconstructive procedure after total laryngopharyngoesophagectomy: the combined use of gastric pull-up and pectoralis major flaps to reconstruct the massive defects resulting from the radical ablative surgery. The pectoralis major flap was either used along with gastric pull-up to restore the intestinal continuity or to cover the neck defects to protect the exposed great vessels and the pharyngogastric anastomoses. The functional and oncologic outcomes from this series of patients suggest that this combined flap technique for reconstruction is a reliable method for patients with advanced SCCHP after radical resection.

To date, many techniques have been reported to reconstruct circumferential pharyngeal defects following total pharyngolaryngectomy, including regional myocutaneous flap, jejunal free flap, fasciocutaneous free flap, and gastric pull-up. The first-line reconstructive method should be a single-stage reconstruction with low morbidity and mortality, short hospital stay, and early restoration of swallowing. Jejunal free flap for reconstruction of circumferential pharyngeal defects has gained popularity with numerous large case series because of its advantages, such as lower perioperative mortality, availability of a ready-made mucosal tube with a lumen caliber closely matching the esophagus, ability to perform an immediate single-stage reconstruction, less gravitational tension on the anastomotic suture lines than a pedicled flap, and rapid return to swallow. However, the major disadvantage of the jejunal flap is the donor-site morbidity. Fasciocutaneous free flaps, including radial forearm free flap and anterolateral thigh flap, also have a lot of advantages, such as feasibility for a 2-team approach, low donor-site morbidity, large caliber, and lengthy vascular pedicle; however, high incidence of fistula and stricture should also be considered.

Although other popular reconstructive methods offer advantages for some patients with SCCHP, gastric pull-up is usually indicated when the primary tumor involves the cervical esophagus or there is a synchronous second primary tumor in the thoracic esophagus that requires esophagectomy [19, 20]. Gastric pull-up may also be considered in other circumstances, such as when donor vessels are not available for a free flap because of radical bilateral neck dissection [16]. However, the gastric pull-up flap has some limitations, as it allows reconstruction only up to the tongue base. As a result, in patients with a small stomach or whose resection extends farther than the level of the tongue base, reconstruction with gastric pull-up alone can cause anastomotic tension that could lead to postoperative fistula. In such patients, therefore, a second flap should be considered to repair the defects together with gastric pull-up to create a tension-free closure.

The pectoralis major flap is one of the most commonly used pedicled flaps for head and neck reconstruction because of its robust blood supply. Moreover, this flap can be used to reconstruct the defects even at the level of infratemporal fossa because of its long pedicle. Thus, the pectoralis major flap is an alternative for head and neck surgeons when 1 flap alone cannot entirely cover the defect. In combination with gastric pull-up to restore intestinal continuity, the pectoralis major flap can ensure a tension-free anastomosis and provide sufficient blood supply for the anastomosis to reduce the likelihood of anastomotic leakage, which is a severe postoperative complication that could lead to necrosis of the gastric flap. When a pectoralis major flap is used to cover the neck to protect the pharyngogastric anastomosis and the exposed great vessels after gastric pull-up alone is used to restore intestinal continuity, the pectoralis major flap can also provide additional tissue that was not irradiated preoperatively to protect patient from fistulas resulting from postoperative radiotherapy.

In the current study, we did not observe any complications from anastomotic leakage in patients whose intestinal continuity was restored with the combined flaps, suggesting that the pectoralis major flap together with gastric pull-up to restore intestinal continuity is a good option for head and neck surgeons when gastric pull-up alone cannot cover the defects. However, two of the patients whose defects were reconstructed with gastric pull-up alone and covered with a pectoralis major flap developed anastomotic leakage, which was probably due to preoperative radiotherapy. Nevertheless, the leakage was resolved using a contralateral pectoralis major flap, which suggests that a secondary reconstruction with a pectoralis major flap and gastric pull-up is also a trustworthy option for defects resulting from complications.

The combined use of a pectoralis major flap and gastric pull-up to restore intestinal continuity has been previously reported. Marks and Steiger reported 3 cases in which the combined pectoralis major flap and gastric pull-up procedure were used for pharyngeal reconstruction, without leakage or fistula [21]. In their study, the inferiorly based flap created from the anterior surface of the stomach was anastomosed to the posterior pharyngeal wall, and the pectoralis major flap was raised to suture to the tongue base and cover the anterior wall of the stomach. However, in our study, we used the pectoralis major flap to reconstruct the posterior pharyngeal wall because the flap can reach as far as the level of infratemporal fossa, and the pulled up stomach was then sutured to the tongue base. As a result, intestinal continuity was restored and a tension-free anastomosis was achieved, without compromising the extent of radical resection. Similarly, To et al. [22] reported a case in which the same method as ours was used to reconstruct massive naso-oropharyngeal and esophageal defects. They reported that the method worked well, and the patient had no postoperative leakage. Therefore, the results of our study and the findings from the other 2 reports of reconstructions using this combined flap suggest that combined use of the pectoralis major flap and gastric pull-up to restore intestinal continuity can ensure a tension-free anastomosis and provide sufficient blood supply for the anastomosis, which could lead to a short postoperative recovery time without anastomotic leakage.

Procedures using the pectoralis major flap with other flaps to cover the neck defect resulting from radical dissection also have been reported. Dubsky et al. [23] reported a series of 8 patients with recurrent SCCHP whose surgical defects were reconstructed using free jejunal transfer covered by a pectoralis major muscle flap. They found that this combined flap for reconstruction achieved a relatively long survival and minor perioperative and postoperative morbidity; however, before the study, they had treated two similar patients with reconstruction of the defects using the free jejunal transfer alone, and some postoperative complications developed, such as skin dehiscences and esophageal stenosis. Their results suggest that the additional use of pectoralis major flap could decrease postoperative morbidity, possibly because the flap provides additional nonirradiated tissue and sufficient blood supply. In our study, 8 of 10 patients whose defects were reconstructed using gastric pull-up covered by a pectoralis major flap healed well; 2 patients developed fistula. However, none developed fistula or other complications after postoperative radiotherapy, suggesting that the use of a pectoralis major flap to cover the neck after gastric pull-up could reduce postoperative complications such as graft failure, fistula formation, and anastomotic stenosis.

Our results showed that the 13 patients in whom the pectoralis major flap and gastric pull-up were used to restore intestinal continuity had a shorter mean swallowing function recovery time than did the ten patients whose defects were reconstructed using gastric pull-up and covered by a pectoralis major flap, but this finding was inconclusive due to the small number of patients in each group. However, we did observe that these 13 patients had a smooth postoperative recovery without anastomotic leakage. This finding suggests that the combined use of pectoralis major flap and the gastric pull-up to restore intestinal continuity is a safe reconstructive method for patients with extensive hypopharyngeal cancer after uncompromised resection. Furthermore, it is probable that using this combined flap technique to restore intestinal continuity contributes to a quick postoperative recovery without severe complications and thus leads to a shorter swallowing function recovery time after surgery. However, future studies with large sample sizes are needed to verify our findings.

The oncologic outcomes of this series of patients are not surprising. Although 5 patients died of disease—likely because of their advanced disease stage and poor pathological grade—the other 18 patients had a disease-free period of at least 1 year with normal swallowing function, and 6 were still alive with good function at the time of analysis. Therefore, despite the short overall survival times for patients with advanced SCCHP, we can speculate that this combined flap technique can help these patients regain some quality of life after radical surgery. Furthermore, more patients with advanced SCCHP might be indicated for radical surgery because the combined flap technique makes reconstruction possible.

## Conclusions

In summary, we have described a successful reconstructive technique using a pectoralis major flap combined with gastric pull-up for patients with advanced SCCHP after total laryngopharyngoesophagectomy. The functional and oncologic outcomes from this study suggest that this combined flap technique is a reliable method for reconstruction for patients with advanced SCCHP after radical resection. Furthermore, as gastric pull-up alone may be insufficient to restore intestinal continuity in patients with a small stomach or whose resections extend farther than the level of the tongue base, the pectoralis major flap might be routinely considered as an additional flap for patients undergoing gastric pull-up after total laryngopharyngoesophagectomy.
